# Expansion and Contraction of the Foot, and How to Shoe It

**Published:** 1882-01

**Authors:** John N. Navin

**Affiliations:** Veterinary Editor of Indiana Farmer


### ART. IV.—EXPANSION AND CONTRACTION OF THE FOOT, AND HOW TO SHOE IT.

(*Continued.*)

In my effort to revolutionize the old-time method of shoeing horses, which has been no more or no less than a necessary evil, by nailing down the elastic foot to an inelastic piece of iron called a shoe, I shall address the general public in the plainest language at my command, using as few technicalities as possible.

Had I the veterinary faculty alone to address, my task would be an easy and pleasant one, for that class, as a general rule, is free from prejudice, and competent to comprehend my entire theory. It is, however, quite different in addressing the general public, those who are the chief owners of a majority of the horses of the country, and w o, as a class, are intelligent enough upon all subjects regarding the horse except his diseases, his anatomy and physiology, and especially are they ignorant of the structure of the foot, and how to shoe it,—an art to which they have paid no attention, and cared as little, whenever a blacksmith could be procured, who could fasten a shoe to stay on six or seven weeks, or until the shoe is carried forward by the natural growth of the foot, and its heels rest upon the sole, or bars inside the wall; this, with the cramping of the foot by the shoe, could scarcely fail of evil results. Under such circumstances, I am not unmindful of the amount of ignorance and prejudice I have to encounter, and the utter impossibility of changing old practices, and removing old prejudices, until the owners of horses are made familiar with the anatomy and physiology of the foot.

With such knowledge, the otherwise intelligent owners of horses will readily become convinced of facts which otherwise would remain wrapped in prejudice in the future as in the past. Indeed, the intelligent farmer, and other owners of horses, are not the classes who offer the greatest resistance to science and progress; but a class of men quacks, who also uneducated, possess an unbounded amount of audacity and impudence, and try to show their own fancied skill by antagonizing every theory which differs from their own shallow ideas.

One assurance I have, however, is that every high-minded, educated veterinarian stands ready to measure my theory with the unerring law of nature, and facts previously known to the profession. This is all I desire or expect; therefore, I shall endeavor to define the anatomy and physiology of the different organs composing the foot, how its expansion and contraction is effected before being shod and after, and how it should be shod, to permit its natural expansion and contraction as fully as when shod as before.

### ANATOMY AND PHYSIOLOGY OF THE FOOT.

Externally, the foot is composed of a wall (hoof), a sole, a frog, two bars, and a frog-ban l, all of which are insensible or destitute of feeling, and are produced by the sensible internal organs out of the blood they contain. Internally, it is composed of a sensible sole, and a sensible frog, a sensible corronary substance, a patella (or coffin-bone), a navecular (or shuttle-bone), two lateral and two inferior cartilages. The perforous tendon, though not considered a part of the foot, enters at the heel, and deserves a passing notice. This is the main tendon of the limb, and is inserted into the inferior surface of the coffin-bone. There are also about five hundred narrow, thin, leaf-like sensible strips attached to the coffin-bone called laminæ, which are dovetailed into a like number of insensible laminæ attached to the internal surface of the wall. By this wonderful dovetailed attachment, the entire weight of the horse is attached to the internal surface of the wall; so that, while standing at rest, but little if any of his weight rests upon the sensible parts of the foot.

Viewing the foot in a standing position, the wall and frog-band are the only organs visible; when the foot is taken up in the act of shoeing, however, the insensible sole, the insensible frog, the bars, and the inferior border of the wall are seen. The above organs, therefore, constitute both the external insensible and the internal sensible organs composing the foot.

In demonstrating the anatomy and physiology of the organs separately, I shall not confound the reader by going into a minute detail or scientific analysis of the parts composing each organ, except so far as it is necessary in proving the cruelty of the present way of shoeing, and the humane results of the new method.

*The insensible frog* is a letter V, or wedge-shaped mass of horny, fibrous substance situated in the inferior posterior portion of the foot; it is partially divided upon its outer surface by a fissure called its cleft, but not to its apex or its base. Both sides of the fissure are called its bulbs, which never come in contact with the wall, except at their extreme posterior termination. It divides the sole in its center, having an equal portion on each side of it Perhaps it might be said that the sole is divided for the reception of the frog, to enable both in conjunction to sustain the weight thrown upon them in violent action. It is firmly attached to the sole on each side where it divides it, leaving a space on each side which is occupied by the bars between it and the wall. In a healthy state, the horny frog is elastic to a high degree; so much so, indeed, that, when violently pressed, it bends under the weight, its bulbs expand, almost reaching the wall, obliterating its cleft, but as soon as relieved of the pressure, bounds back into its natural shape. I have made many experiments with artificial pressure, and never could force it against the wall.

The extensive uses nature demands from the frog are: To defend the sensitive frog above it from injury, and to support the weight thrown upon it. It had been erroneously considered that the latter duty had been performed by its contact with the ground; but this opinion will not hold good when we consider that it performs equally well when elevated from the ground by thick, or calked, heeled shoes; in such cases, its junction with the sole supports it equally as well.

*The insensible sole* is placed in the anterior, inferior portion of the foot, and, like the horny frog, is horny, fibrous and elastic, but not as elastic as the frog. It is an arch of greater or less concavity, and is found to differ much in concavity in horses of the same breed, but is much more marked as between fine-bred horses and those of coarser breeds. Its base, or margin, is firmly attached to the inferior margin of the wall all round, a little above its contact with the ground. This greatly increases its concavity, and will be alluded to further on. It is also firmly attached to the frog on each side, where that organ divides it.

*The bars* are an inflection or turning in of the extreme posterior ends of the wall upon the sole, in the space between the wall and frog, forming something of a V-shaped eminence upon the sole. Its apex, however, is interrupted by that of the frog, the bars not reaching as far forward as the apex of the frog. The object of nature, in this regard, appears to be two-fold—first, to strengthen the sole where it is divided by the frog; secondly, to perform the office of stays or braces, to resist contraction of the wall upon the internal sensible parts it envelops.

*The sensible sole* is a tough, elastic, fibrous, vascular substance and highly sensible. It is placed above the insensible sole and beneath the coffin bone, to whose laminated edges it is attached all round its inferior border, and serves as an elastic cushion between it and the insensitive sole beneath it, thereby obviating concussion in the unshod foot, but in the foot deprived of expansion is liable to concussion at any time. Its superior surface is composed of a ligamentous, tendinous structure, and its inferior surface presents a cuticular structure liberally supplied with a network of blood-vessels, many of which penetrate the insensible sole, and may be readily seen bleeding in cutting down an injured or diseased sole, and if cut entirely away, a new sole will be furnished by the network of blood-vessels above mentioned.

*The sensible frog* is also a tough, elastic mass, partly ligamentous and partly tendinous and fibrous. It is liberally supplied with nerves and blood-vessels, the latter forming a network. It is situated above the insensible frog, in the posterior part of the foot, occupying the entire space above the insensible frog, to which it fits exactly, and reaches to the cartilages on each side. The perforans, or flexor tendon, alluded to before, passes over it and beneath the navicular bone on its way to its insertion into the inferior surface of the coffin bone. It is thicker than the sensible sole, and irregular in shape. Its uses will be explained further on in the proper place. Doubtless the reader will readily perceive that the sensible sole and frog compose an elastic cushion, or pad, for the reception of the coffin and navicular bones in their descent, when great weights are carried, or the foot is put down heavily in violent action, thus obviating concussion between them and the horny sole and frog beneath them.

*The coffin and navicular bones.*—The pedal, or coffin bone, is the lower bone of the limb. It is composed of a body and two wings, the body anteriorly and the wings posteriorly within the wall, to which it is attached all round, its anterior and latteral surfaces by the laminæ heretofore spoken of. Its inferior concave surface rests slightly upon the superior convex surface of the sensible sole while standing still, but heavily in violent action. As before remarked, the sole is attached to its inferior border all round. Its wings reach backward from its body, and the navicular bone is placed between them and above the sensible frog. The inferior surface of the navicular is quite smooth, and acts as a pulley to the perforous tendon as it passes beneath it and above the sensible frog on its way to its attachment into the inferior surface of the coffin bone, as before alluded to.

Both the coffin and navicular bones are fitted upon their superior surfaces for the reception of the coronary or inferior pastern bone. The coffin bone is supposed to bear two-thirds and the navicular bone one-third of the weight thrown upon the limb, and the junction of the three bones constitutes the coffin joint.

The reader, therefore, will readily understand that the tendon passes beneath the navicular bone and above the sensible frog, so that the tendon bears the navicular and one-third the weight of the limb supported by the sensible frog, and it by the insensible frog. Therefore it is not difficult to comprehend nature's object in placing two such elastic organs in support of the main tendon, and the foolishness of those who claim that the frog is placed there as a wedge to expand the wall.

Suppose, for one moment, some wedge-shaped substance placed between the heels of sufficient density to force the wall apart. The effect upon the sensible parts above it would be, of course, concussion, inflammation and destruction.

*The lateral cartilages* deserve no more than a passing notice, having but little to do with the expansion and contraction of the foot. They, however, are susceptible of great injury from contraction of the wall upon them and other causes. They are situated upon the superior margin of the wings of the coffin bone, and have much to do in the suspension of the limb. The laminæ alluded to upon the coffin bone reach back upon their outer surface. They are, therefore, united to the wall, and assist the coffin bone in its support. Rising above the wings of the coffin bone, to which they are firmly attached, they fill the space above the wings, which without them would be a hollow, empty space. The part they take in the attachment of the coffin bone to the wall, and preventing a latteral or rocking motion of the internal parts, seem to be their chief uses. By their elasticity they readily yield to sudden contraction and expansion of the wall.

*The inferior cartilages* are more elastic than the lateral. Being of fibrous construction, their principal use is to fill up the space between the perforous tendon and the wings of the coffin bone.

*The sensible laminæ* are narrow strips or plates attached to the coffin bone, and latteral cartilages upon their external surfaces from their superior to their inferior margins. They are fibrous in structure, highly sensible and elastic, plentifully supplied with nerves and blood-vessels, and much resemble the pinching up of a lady's frill. They are about five hundred in number. I shall fully describe them in their proper place.

*The insensible laminæ* are equal in number with the sensitive laminæ. Their structure is horny. They are insensible, having neither nerves nor blood vessels, and closely resemble the little leaves upon the inner surface of a mushroom. They and the sensible laminæ are dovetailed into each other, by which union the coffin bone is so firmly attached to the internal surface of the wall that no action of the horse under reasonable circumstances is able to sever them. As before remarked, by this dovetailed union the entire weight of the horse is sustained. An idea of the strength of their union may be gained by seeing a horse jump over a wall five to six feet high, carrying on his back a man weighing one hundred and sixty pounds or over.

*The coronary substance*, commonly called secreting substance, is placed above the coffin bone and within the superior surface of the wall, which it secretes and supplies as fast as worn down or cut away by the smith. About its structure and its functions, however, there exists a great difference of opinion among eminent writers. Some contend that it is a continuation of the inner and middle layers of the skin, while others contend that it is an exclusive organ secreted by the skin and differing from it in structure, but that the skin furnishes it with nerves and blood vessels, and that only a portion of the wall is secreted by it, and that the other portion and the insensible laminæ are secreted by the sensible laminæ. This I deny, and expect to prove further on.

*The coffin bone*, as before remarked, is situated in the anterior cavity of the foot within the wall. It is the last or lower bone of the limb. It is concave on its inferior surface, exactly corresponding with the convex or arched surface of the sole, and on its lateral and anterior surfaces exactly corresponds to the concave form of the wall to which it adheres closely. It is an exceedingly light bone; its exterior surface is quite rough and ariolar, for the attachment of its periostial membrane, much resembling the attachment of the capsular ligaments to jonts; but, unlike the bones of joints, it is furnished with little grooves in which nerves, absorbants and blood vessels repose and are protected. Its body is internally traversed with holes or passages, through which blood vessels and nerves pass on their way to their destination. Thus it will be seen that they are protected, as by an arch, from the injury they would otherwise sustain by passing beneath the bone. As before remarked, the perforous tendon is inserted into its inferior surface.

It is covered with a double, tough membrane, similar to the periosteum of other bones of the body; but that upon the coffin bone differs from the other in so far as that the covering of the coffin bone is double, composed of two layers of membrane, the inner layer closely attached to the bone, and the outer, which is much the widest, is thrown into folds or plaits, and receive the nerve laminæ. Those are the sensible laminæ before mentioned. Mr. Brunal says that the supposed elasticity attributed to the plaits is derived from the substance which connects the two layers together, and furnishes a bed for the blood vessels and nerves which proceed through the ariolar structure of the bone.

The entire periostial covering of the bone composing both the layers and laminæ appear to be produced by the coronary substance, for the outer layer, which pinches up into folds forming the sensitive laminæ, seems to be a continuation of the cuticular covering of the coronary substance, which is much greater in circumference than the coffin bone is. This, to a great extent, accounts for its being thrown into folds.

*The wall, or hoof,* is the horny covering designed by nature for a protection to the internal sensitive parts it envelops, and so completely is it organized and shaped, that not only does it defend the internal organs of locomotion, but also the coronary substance which produces it within its superior border, and with its inferior border defends the outer insensible sole to a certain extent from injury, by reaching below it and first coming in contact with the ground, the better enabling it to support the entire weight of the animal. Its border suffers much wear and tear, hence the necessity for shoeing. The wall gradually decreases in height to the heel, and if continued in a direct line backward, would end in two sharp points, but instead they become inflected downward, inward and forward upon the sole, and are those before spoken of as the bars.

*Form and formation of the wall.*—Its superior border, commonly called its coronary border, is scooped, or beveled out upon its internal superior surface, to about three-fourths of an inch around its entire circumference. It rapidly becomes thinner toward its superior margin, until its extreme edge becomes acute. This is named its coronary concavity (coronary ring). Within this concavity reposes the coronary substance which secretes the wall. Some contend that only a portion of the wall is secreted by it, which I deny. Upon the coronary substance may be seen with the natural eye an indefinite number of villi, or small projections, resembling papillæ; and upon the beveled surface of the wall a like number of perforations or depressions, which receive each one of the villa of the coronary substance. These perforations are the superior or upper ends of so many cylindrical little hollow tubes, which reach to the inferior margin of the wall, and are the basis of its structure.

If nature had not provided means for the connecting or gluing of these little tubes together, the horse would be seen standing upon thousands of little stilts, which would, more than anything else, remind one of a brush with the hairs toward the ground. Each villus is seen to dip into one of those little tubes, which they secrete and supply as fast as they are worn down or cut off at its ground surface Nature, however, comes to the rescue, and furnishes a cementing substance which binds them together and obliterates the spaces between the tubes, making it a solid wall as we see it. The substance which connects the tubular structure is called its cementing matrix, and about its secretion there has arisen a perfect Babel of different opinions. One gentleman pretends to prove, with seeming force, that the connecting matrix and insensible laminæ upon the internal surface of the wall are secreted by the sensible laminæ upon the coffin-bone which is dovetailed into them. Another gentleman says that the matrix is secreted by the coronary substance which secretes the little tubes also, but that the sensible laminæ secrete the insensible laminæ. Two other eminent writers say that the tubular structure—the connecting matrix and insensitive laminæ—are all secreted by the coronary substance. I say so too, and expect to fully prove it.

Hoping that the intelligent reader fully understands my plain and concise definition of the organs composing the foot, I shall proceed to not only show, but prove, first, that the wall of the unshod foot expands under violent pressure, and contracts to its natural dimensions as soon as relieved of such pressure; secondly, that the frog, exercises but very little part in expanding the shod foot; and, thirdly, none at all in expanding the shod foot; fourth, that expansion is necessary to the healthy condition of the foot, shod and unshod; fifth, that the insensible sole is the chief organ which directly expands the wall, with, of course, the agency of other organs; sixth, that the present method of shoeing is barbarous; seventh, that the present shoe in use is, by its first application, indirectly if not directly injurious by sowing the seeds of future disease in at least the feet of horses used in quick action. It is true that horses used in slow action and not too heavily burdened may escape injury for an indefinite time, perhaps a few for life, without marked injury; but those ridden or driven rapidly upon hard surfaces are certain to receive injury, sooner or later. Scarcely is a horse used in quick action that is not the victim, more or less, of the old method of shoeing. The fact that every horse one meets is not lame and limping, is no proof at all that they are not suffering—both feet being equally affected, they cannot limp on either. For if a man stands at a crossing of two paved or macadamized streets, scarcely can he notice a horse trotted by him who throws his feet down independently, and with a bold loose step, both feet being equally cramped. He does not limp on either, but ambles along, putting his fore-feet down as lightly as possible, and trying to keep his hind feet as far under his body as he can.

In proof of the above, I propose to take a four, or a three-year-old colt that never has been shod, and put one of the common shoes upon one of his feet, leaving the other bare, turn him on pasture, keep the same foot continually shod for six or twelve months, then put him to work upon hard surfaces, and he will be almost certain to limp on the shod foot, more or less, if driven fast. Especially will he limp if a shoe, nailed only on the outside, with one nail near the toe on the inner side, is put on the foot left unshod until then. This I know, having been experimenting upon the subject.

Again, take a horse having both fore-feet shod, and both contracted and corned, take off one shoe, and leave the other one on, work him at a plow and harrow, on dry ground, in dry weather. The unshod foot will be certain to get well, and the foot wearing the shoe will be no better, but will be most certain to grow worse.

Still further, if a horse has corns in both feet, and an expansion shoe is put upon one, and the common shoe on the other, the foot wearing the expansion shoe will expand and get well, and the other is certain to grow worse. This I have also experimented on both shoes.

In proving the above seven propositions, I shall call the reader's strict attention to the following simple fact, which I shall prove the entire seven by:

Take a sound-footed horse, cut his frog down as low as possible, so low that no action of his can make the part left untouched reach the ground, then put on a bar shoe, whose bar completely covers the frog, and so concaved that the frog never could reach it. Now, if the shoe is nailed only on its outside arm to the foot, and one nail at the toe on the inner side, put the horse to quick action for three or four weeks, and it will be found that the inner arm will be polished bright, and more or less elevated at the heel.

This proves at once the seven propositions: First, that the wall expands and contracts in quick action. Second, that expansion is necessary, else it would not expand. Third, that the frog has no possible agency in its expansion. Fourth, if the foot is nailed on both sides back to the heels, it cannot possibly expand. Fifth, that whereas the frog has no part in expansion, the sole is the only means of expanding the wall, by yielding under the weight thrown upon its crown, it expands at its base, and forces the wall outward. Sixth the above being facts uncontrovertible, the old shoe which prevents expansion is barbarous in its application, and consequently sows the seeds of disease, more or less, by its first application.

If the above are stubborn facts, which they are, the next inquiry presenting itself is, what kind or form of shoe is best adapted to the safety and healthfulness of the foot. After all that has been written above, and the experience of intelligent veterinarians during this, the nineteenth, century, the answer is anticipated—a shoe which, defending the foot from wear and tear, also permits it to expand and contract as readily as does the unshod foot. With such a shoe in use, shoeing is no longer a necessary evil, as in the past, but a boon of mercy to the noble, useful servant of man.

From the fact that all men cannot comprehend science alike and, as remarked in advance, old habits and prejudices blind thousands of men's eyes and bind them to old theories to such a degree that I feel the necessity of proving beyond controversy that the sole is the organ that directly expands the wall, and that proves all that is necessary to prove or can be proven.

In proving this I am compelled to draw the reader's attention back to two facts; first, when the frog is elevated from the ground by cutting it away, or by thick shoes or high calkings, or shod with bar shoes, as described, the frog cannot possibly expand the wall.

If the reader still cannot comprehend and wishes further explanation, I take him back to the anatomy and physiology of the parts, and remind him of the many times I promised to further call his attention to the anatomy of the parts, and remind him that the sole is more or less concave or arched, and that it is firmly attached to the wall, all round its inferior border, a little above its ground surface (margin); that the sole is elastic and the wall slightly so; that the coffin bone is attached to the wall by elastic laminæ, dove-tailed into a like number upon the wall; therefore, can anything either in mechanism or nature more fully prove that, when weight is thrown heavily upon the coffin bone, the elastic laminæ extend and permit the coffin bone to ascend upon the sole, that organ being arched and elastic, readily recedes (sinks) under the weight from above, and in so doing, forces the wall outward at its base, and relieves itself of the pressure thrown upon its crown, and obviates concussion. The navicular bone also descends upon the frog at the same time, that organ being highly elastic, also yields to the pressure upon it, but neither in shape or density has it the power to expand the foot —either of the unshod or shod foot—especially while elevated from contact with the ground. Its firm junction with the sole and its great elasticity is the only means by which it is able to give partial support to the perforous tendon and navicular bone, and to descend beneath the same pressure simultaneously with the coffin bone, the sole, on account of its arched form and partial elasticity, and the frog, by its great elasticity and its junction with the sole.

JOHN N. NAVIN, V.S.,
*Veterinary Editor of Indiana Farmer.*

(*To be continued.*)

